# Supplementary material for: gUMI-BEAR, a modular, unsupervised population barcoding method to track variants and evolution at high resolution
Source: PLoS One. 2023 Jun 7;18(6):e0286696. doi: 10.1371/journal.pone.0286696 (PMC10246843; doi:10.1371/journal.pone.0286696)
Supplement: S1 Fig — gUMI region of the donor DNA prior to transformation (upper panel) and DNA from lineages participating in the experiment (lower panel) were extracted and deep-sequenced to reveal the nucleotide composition for each position in the gUMI (x-axis). The overall height of each base is proportional to its probability (y-axis). Colours represent different nucleotides. (DOCX) [file pone.0286696.s001.docx]

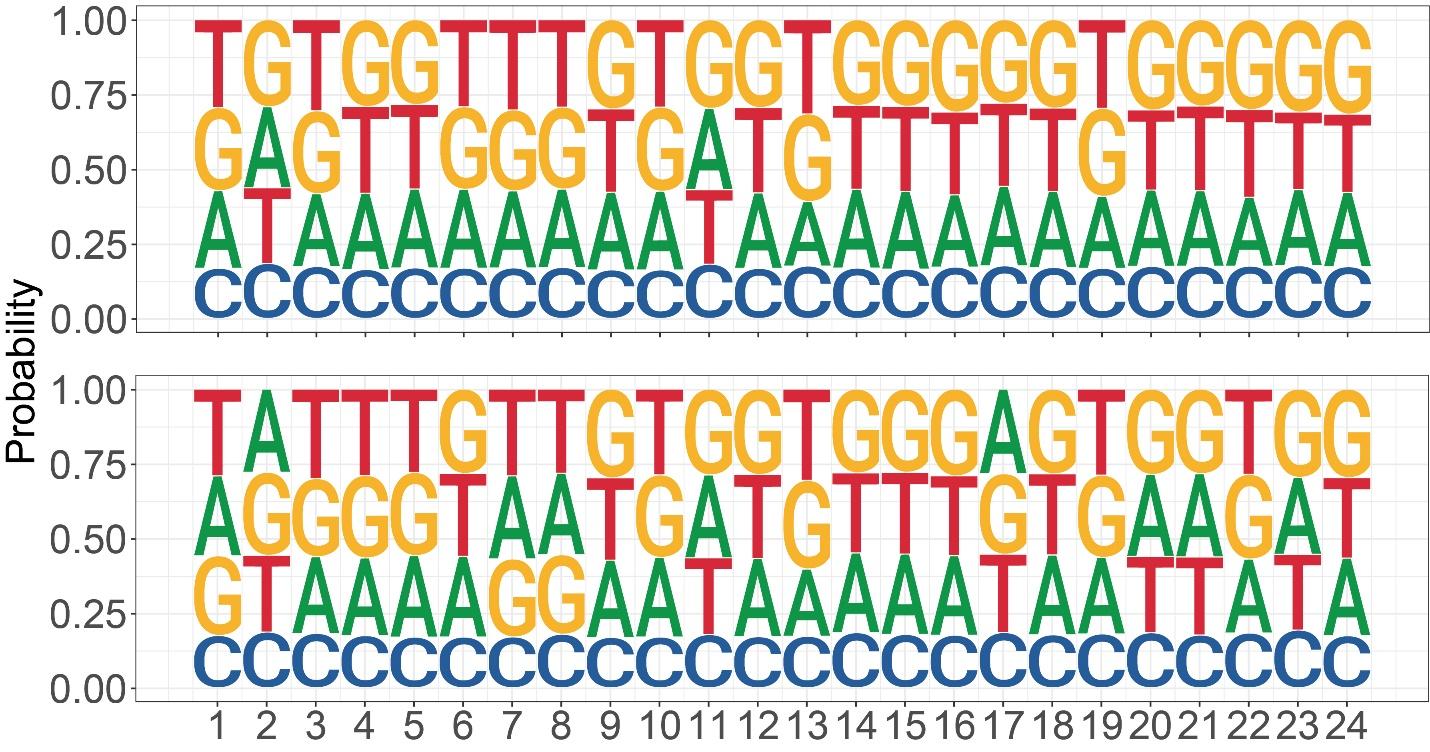


**S1 Fig Nucleotide composition by position for the gUMI from donor DNA and lineages**

gUMI region of the donor DNA prior to transformation (upper panel) and DNA from lineages participating in the experiment (lower panel) were extracted and deep-sequenced to reveal the nucleotide composition for each position in the gUMI (x-axis). The overall height of each base is proportional to its probability (y-axis). Colours represent different nucleotides.
