# Supplementary material for: gUMI-BEAR, a modular, unsupervised population barcoding method to track variants and evolution at high resolution
Source: PLoS One. 2023 Jun 7;18(6):e0286696. doi: 10.1371/journal.pone.0286696 (PMC10246843; doi:10.1371/journal.pone.0286696)
Supplement: S2 Fig — GC content was measured based on sequences derived from all lineages at three steps during library construction. (left, red) Donor DNA prior to transformation, (middle, green) from lineages at the beginning of the experiment, (right, blue) from lineages at the end of the experiment. All data are presented in histograms to reveal the GC content distribution in the populations. (DOCX) [file pone.0286696.s002.docx]

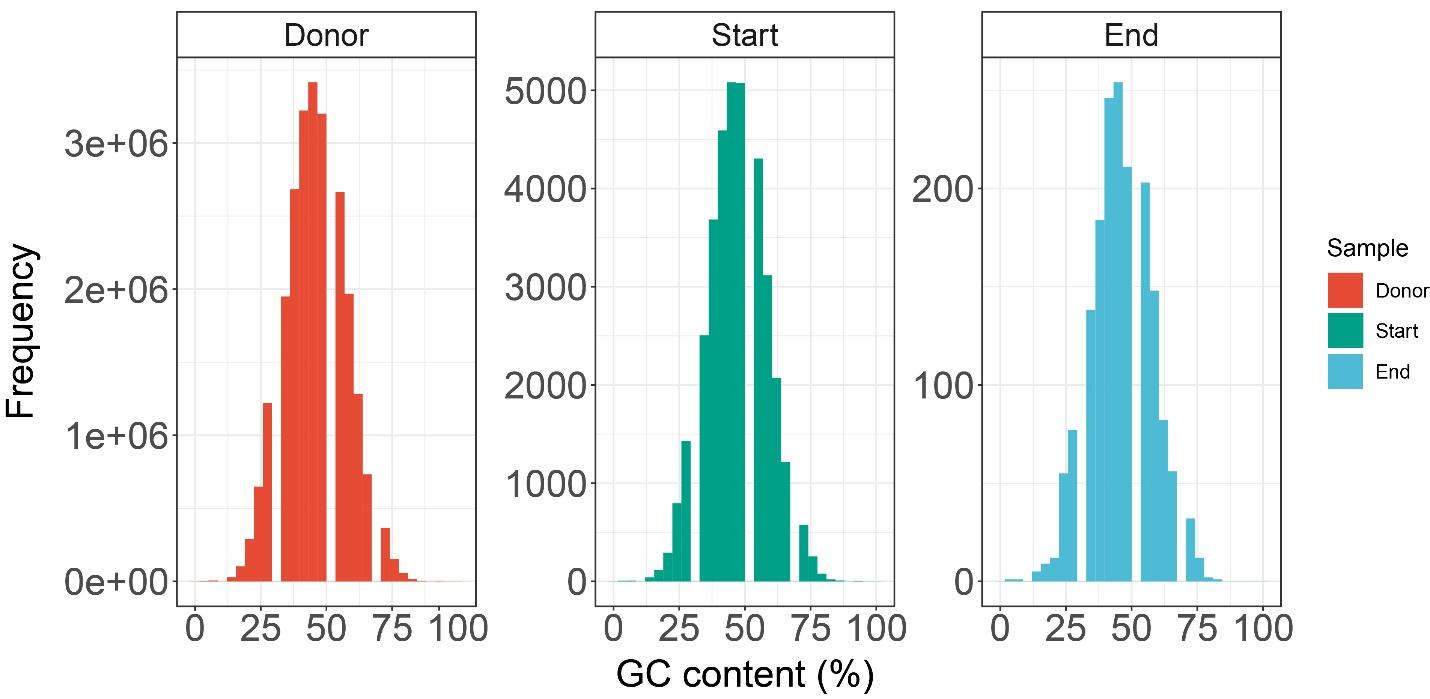


**S2 Fig Guanine-Cytosine content distributions for the gUMI from three stages in the experiment with no initial fitness variations**

GC content was measured based on sequences derived from all lineages at three steps during library construction. (left, red) Donor DNA prior to transformation, (middle, green) from lineages at the beginning of the experiment, (right, blue) from lineages at the end of the experiment. All data are presented in histograms to reveal the GC content distribution in the populations.
