# Supplementary material for: gUMI-BEAR, a modular, unsupervised population barcoding method to track variants and evolution at high resolution
Source: PLoS One. 2023 Jun 7;18(6):e0286696. doi: 10.1371/journal.pone.0286696 (PMC10246843; doi:10.1371/journal.pone.0286696)
Supplement: S3 Fig — Doubling time for the whole culture was calculated based on dilution rate of the turbidostats and is presented on a log scale (left y-axis, orange line) and plotted as function of time. Glucose concentrations were measured for each sample taken (right y-axis, blue line). The experiment started at an optimal growth temperature of 30°C that was altered to induce stress as noted by the dotted lines. (DOCX) [file pone.0286696.s003.docx]

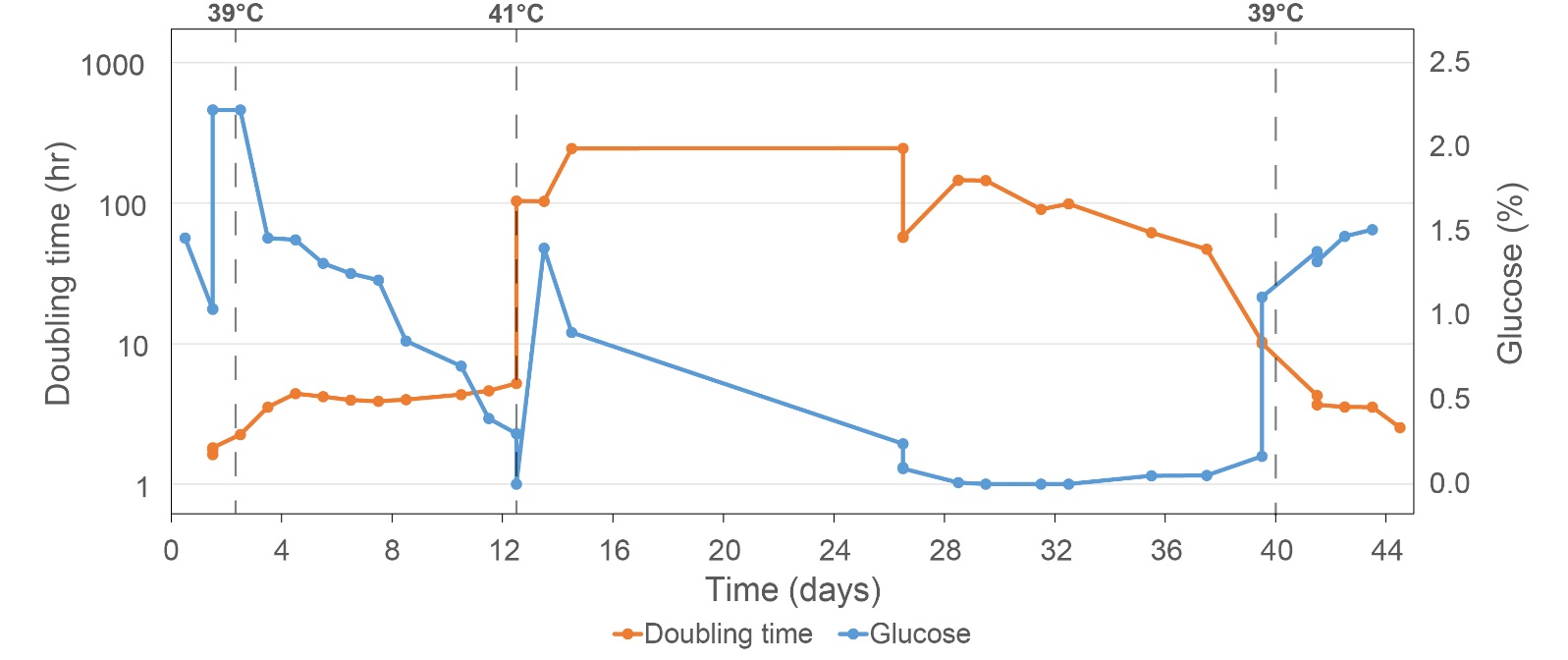


**S3 Fig Mean doubling time and glucose concentration for the experiment in population exhibiting no initial fitness variations**

Doubling time for the whole culture was calculated based on dilution rate of the turbidostats and is presented on a log scale (left y-axis, orange line) and plotted as function of time. Glucose concentrations were measured for each sample taken (right y-axis, blue line). The experiment started at an optimal growth temperature of 30°C that was altered to induce stress as noted by the dotted lines.
