# Supplementary material for: gUMI-BEAR, a modular, unsupervised population barcoding method to track variants and evolution at high resolution
Source: PLoS One. 2023 Jun 7;18(6):e0286696. doi: 10.1371/journal.pone.0286696 (PMC10246843; doi:10.1371/journal.pone.0286696)
Supplement: S4 Fig — The kernel density estimations of the Shannon entropy for nucleotide substitutions in the HSP82 ORF in the genenmorph amplicon after in vitro mutagenesis (black) and three replicates of the assembled donor DNA (green, red and blue) are shown. Three peaks can be observed—first at entropy 0, in which all substations were to the same nucleotide, second at entropy 1, in which substitutions were equal between one of two possible substitutions, and finally, the third peak at entropy 1.5 in which an equal probability for all mutations is observed. The donor DNA distribution mimics the one produced by the genemorph, indicating that no bias was induced during donor construction. (DOCX) [file pone.0286696.s004.docx]

**
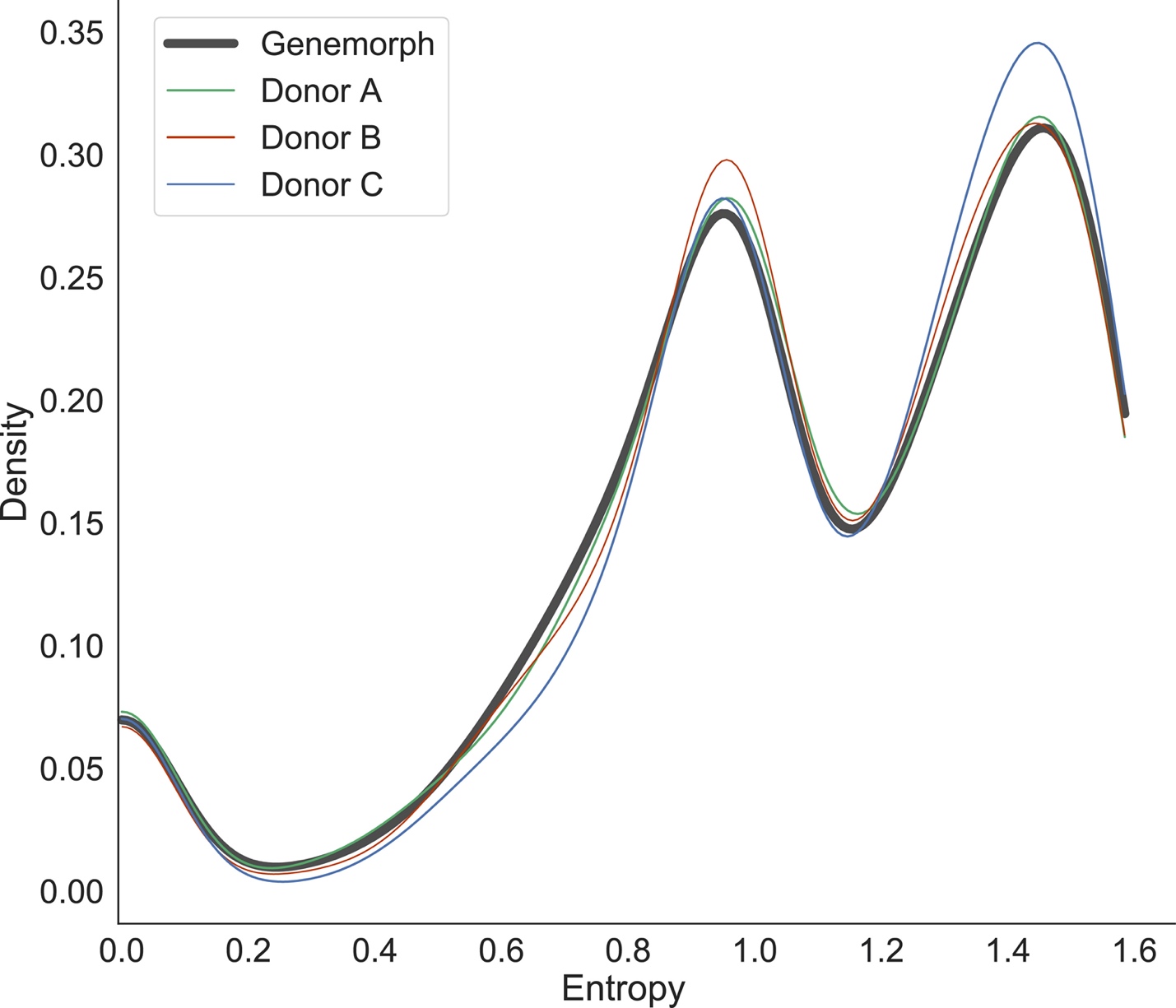
**

**S4 Fig Entropy kernel density estimations for the *HSP82* ORF before and after incorporation into the gUMI-box**

The kernel density estimations of the Shannon entropy for nucleotide substitutions in the *HSP82* ORF in the genenmorph amplicon after *in vitro* mutagenesis (black) and three replicates of the assembled donor DNA (green, red and blue) are shown. Three peaks can be observed - first at entropy 0, in which all substations were to the same nucleotide, second at entropy 1, in which substitutions were equal between one of two possible substitutions, and finally, the third peak at entropy 1.5 in which an equal probability for all mutations is observed. The donor DNA distribution mimics the one produced by the genemorph, indicating that no bias was induced during donor construction.
