# Supplementary material for: gUMI-BEAR, a modular, unsupervised population barcoding method to track variants and evolution at high resolution
Source: PLoS One. 2023 Jun 7;18(6):e0286696. doi: 10.1371/journal.pone.0286696 (PMC10246843; doi:10.1371/journal.pone.0286696)
Supplement: S1 Table — Seven different mini-libraries were prepared while varying the following parameters: No. transformations—the factor applied to the volumes given in the protocol published by Ryan et al.1, namely, 1 μg pCAS, 5 μg donor DNA, and 90 μL competent cells. Hence, No. of transformations = 10 indicates the use of 10 μg pCAS, 50 μg donor DNA, and 900 μL competent cells; Tsel, selection time, being the number of hours the library was grown for after transformation and before sorting; No. cells sorted, total number (in thousands) of viable cells sorted following staining with propidium iodide and selection (see Methods); Texp, expansion time, being the time (hours) the library was allowed to expand before division into 100 aliquots; No. unique lineages, number of viable lineages in each mini-library as determined by deep-sequencing. (DOCX) [file pone.0286696.s005.docx]

| Mini-library Name | No. transformations | T_sel_ (h) | No. cells sorted (×10^3^) | T_exp_ (h) | No. unique lineages |
| --- | --- | --- | --- | --- | --- |
| B2 | 10 | 20 | 55 | 31 | 5349 |
| B3 | 10 | 20 | 55 | 31 | 4865 |
| B10 | 10 | 20 | 10 | 31 | 1636 |
| C55 | 1 | 20 | 55 | 31 | 2768 |
| C10 | 1 | 20 | 10 | 31 | 1538 |
| D55 | 10 | 21 | 55 | 34 | 5189 |
| D110 | 10 | 21 | 110 | 34 | 9028 |

**S1 Table Mini-library parameters**

Seven different mini-libraries were prepared while varying the following parameters: No. transformations - the factor applied to the volumes given in the protocol published by Ryan et al.^1^, namely, 1 µg pCAS, 5 µg donor DNA, and 90 µL competent cells. Hence, No. of transformations = 10 indicates the use of 10 µg pCAS, 50 µg donor DNA, and 900 µL competent cells; T_sel_, selection time, being the number of hours the library was grown for after transformation and before sorting; No. cells sorted, total number (in thousands) of viable cells sorted following staining with propidium iodide and selection (see Methods); T_exp_, expansion time, being the time (hours) the library was allowed to expand before division into 100 aliquots; No. unique lineages, number of viable lineages in each mini-library as determined by deep-sequencing.
