# Supplementary material for: gUMI-BEAR, a modular, unsupervised population barcoding method to track variants and evolution at high resolution
Source: PLoS One. 2023 Jun 7;18(6):e0286696. doi: 10.1371/journal.pone.0286696 (PMC10246843; doi:10.1371/journal.pone.0286696)
Supplement: S1 File — (DOCX) [file pone.0286696.s006.docx]

**Supplementary results**

**Proteomics analysis of Hsp82 variants**

Proteins often carry out their functions through interactions with other proteins to form multi-protein complexes. Such protein-protein interactions play a central role in various aspects of the structural and functional organisation of the cell, and their elucidation is crucial for a better understanding of biological processes.

To elucidate the role of the mutations found in the final population in our experiments in their ability to overcome other variations, we analysed their involvement in the dimer interface.

The crystal structure of the *Hsp*82 homodimer from *Saccharomyces cerevisiae* is known (PDB-ID 2CG9).  The *Hsp*82 structure is missing two segments ^217^V-K^261^ and the C-terminal ^678^I-D^709^. To gain insights into the structural role of the different *Hsp*82 mutations we utilize the deep learning algorithms implemented in the AlphaFold2 software (Google/DeepMind’s artificial intelligence system^2^). The AlphaFold2-multimer was used to predict the *Hsp*82 homodimer of all 28 different constructs, obtained by Loop genomics long read sequencing, each containing between 1 to 4 different mutations and some with deletion of the C-terminal segment (^701^ADTEMEEVD^709^). The different constructs contain 50 different mutations (I20T, A87T, L127I, N164K, V201M, D236E, E250D, K258I, K337E, K342, D352Y, E353V, N377I, N405I, A438V, Y445H, K449M, D459N, K480Q, I505V, K514E, K514R, T525A, D534Y, P549S, V566G, A574T, R591K, M593V, A595V, L598M, M603I, K618E, K626E, K640R, F664Y, V699I , V699D, P700D, A701, D702E, T703S, E704D, M705K, E706, E707, V708, and D709). The WT *Hsp*82 homodimer model (residues 1-709) is shown in cyan and purple, and all mutations found by long-read sequencing are shown as blue and pink balls for the two *Hsp*82 monomers, respectively (Fig 4c). The dimer interface of the various constructs of the *Hsp*82 model contains between 130-260  contacts up to 3.5 Å involving 60-80 different amino acid residues. Approximately half of them appear in the dimer interface of each of the 28 different constructs and 8 are involved in salt bridge contacts (E4-K86, R380-E381, K423-D600, and K484-E660).  Only 4 of the residues involved in the dimer interface are mutated residues found in the following constructs; I505V (*Hsp*-11), A595V (*Hsp*-4), M603I (*Hsp*-20), and L598M (*Hsp*-29). Since the dimer interface of each of the *Hsp*82 constructs involves between 60-80 different residues, it seems reasonable to assume that a single point mutation in a residue involved in the dimer interface will not prevent dimer formation.

**Supplementary methods**

**Restriction Free (RF) cloning and guide-RNA design**

For both applications of our method, we used Chop-Chop^3^ (CHOPCHOP (uib.no)) to find the best matching sequence in our locus of interest for Crispr/Cas9 double-strand breakage.

For the experiment with the population exhibiting no initial fitness differences, we made a single double-strand breakage using the following gRNA sequence:

5’-AGAGCGTCAATCAAGAAAG-3’

and the resulting primers for RF cloning to the pCAS vector:

5’-CGGGTGGCGAATGGGACTTTTAGAGCGTCAATCAAGAAAGGTTTTAGAGCTAGAAATAGC-3’

5’-GCTATTTCTAGCTCTAAAACCTTTCTTGATTGACGCTCTAAAAGTCCCATTCGCCACCCG-3’

For the use in tracking *HSP82* gene variants, we induced two double-strand breaks to integrate our donor DNA into the locus of interest. Two pCAS vectors were cloned using the following gRNA sequences:

Upstream to the *hsp82* gene: 5’-CAAACAAACACGCAAAGATA-3`

Downstream to the *hsp82* gene: 5’-AGCTGACACCGAAATGGAAG-3'

and the resulting primers for RF cloning to the pCAS vector:

Upstream of the *hsp82* gene:

5’-CGGGTGGCGAATGGGACTTTTCAAACAAACACGCAAAGATAAAAGGTTTTAGAGCTAGAAATAGC-3’

5’-GCTATTTCTAGCTCTAAAACTATCTTTGCGTGTTTGTTTGAAAAGTCCCATTCGCCACCCG-3’

Downstream of the *hsp82* gene:

5’-CGGGTGGCGAATGGGACTTTTAGCTGACACCGAAATGGAAGAAAGGTTTTAGAGCTAGAAATAGC-3’

5’-GCTATTTCTAGCTCTAAAACCTTCCATTTCGGTGTCAGCTAAAAGTCCCATTCGCCACCCG-3’

**Donor construction for tracking *HSP82* variants**

The donor DNA construct was assembled in stages, via six PCR reactions that constructed and then assembled two sub-constructs as described in the “Results” section. To build the first sub-construct, the HSP82 gene was amplified from the genome of the BY4741 yeast strain (forward primer 100 bp upstream of gene - Genemorph_F; reverse primer 100 bp downstream of gene - Genemorph_R ; PCR 1: kappa 50 μL, annealing at 60 °C, 25 cycles, 2 min elongation time, 90 ng genomic DNA template). Random mutations were inserted using the GeneMorph II random mutagenesis kit using the Genemorph_F and Genemorph_R primers (1000 ng of template amplicon and 30 cycles to ensure a low mutation rate of 0–4.5 mutations/kb, PCR 2: annealing at 60 °C, 4 min elongation time). Two overhangs were added to the mutated gene by a single PCR reaction. One overhang (LHA primer) contained a 75 bp sequence upstream and a 5 bp sequence downstream of the integration site. The second overhang (HSP82 _Read1) comprised a 15 bp sequence upstream of the stop codon of the genomic HSP82 gene and a Read-1 binding site for Illumina NGS platforms (PCR 3: kappa 50 μL, annealing at 60 °C, 25 cycles, 2 min elongation time, 40 ng mutated HSP82 template).

The second sub-construct was made by adding two overhangs. One primer (Gumi2Hyg) contained: a binding site for Read-1; not G nucleotide (5×H); the gUMI barcode comprised of a 24 bp random sequence, a Linker sequence, and a 25 bp sequence complementary to the Hygromycin B resistance cassette as found on the vector pAG32. The second primer (RHA) contained a 70 bp sequence downstream of the stop codon site of the gHSP82 gene and a 26 bp sequence upstream of the stop codon of the Hygromycin B cassette (PCR 4: kappa 50 μL, annealing 60 °C, 25 cycles, 2 min elongation time, 10 ng pAG32 template).

Following PCR cleanup, the full donor DNA construct was assembled by overlapping the two sub-constructs at their Read-1 regions in a PCR reaction that used PrimeSTAR GXL DNA Polymerase (PCR 5: 50 μL annealing at 60 °C, 15 cycles, 4 min elongation time, 10 ng of each construct, no primers). The donor DNA construct was then amplified using a 5 µL aliquot of the product of PCR 5 (primers, LHA & RHA; PCR 6: kappa 50 μL, annealing at 60 °C, 15 cycles, 4 min elongation time). Primers were obtained from IDT (Israel), with the exception of the RHA primer, which that was obtained from Sigma (Israel).

**All primer sequences used for the donor construction process**

**Tracking evolutionary dynamics experiment**

Gumi2Hyg******

5’-AAATAGGGGAATGAACGCATATTGGTTTCATTATAGAGCGTCAATCAAGATCGTCGGCAGCGTCAGATGTGTATAAGAGACAGHHHHHNNNNNNNNNNNNNNNNNNNNNNNNTTGGAAGTGTGGCTAGACATGGAGGCCCAGAATACCC - 3’

LHA:

5’ – AGCGTTCCTAGCCCTACCGAGAAATGTGCGTTTATAGTTTGGTGTCTCTTCAGTATAGCGACCAGCATTCACAT - 3’

***HSP82* gene variants**

Genemorph_F

5’ - GTGACCTCCTCATTTCTTCCCG - 3’

Genemorph_R

5’- GTGACCTCCTCATTTCTTCCCG - 3’

LHA

5’ - TGTATTAGAGTTCAAGAAATCATACCTGATAGAAAATAGAGTCCTATAAACAAAAGCACAAACAAACACGCAAAGATATG - 3’

HSP82 _Read1

5’ - CTGTCTCTTATACACATCTGACGCTGCCGACGACTAATCTACCTCTTC - 3’

Gumi2Hyg 5’ –TCGTCGGCAGCGTCAGATGTGTATAAGAGACAGHHHHHNNNNNNNNNNNNNNNNNNNNNNNNTTGGAAGTGTGGCTAGACATGGAGGCCCAGAATACCCTCC - 3’

RHA 5’ –TTATTCATTCGAATACCTATACGTTATATTATGTTTTGTTTATAACCTATTCAAGGCCATGATGTTCTACCAGTATAGCGACCAGCATTCACATAC - 3’

**Supplamentary References**

1. Ryan, O. W., Poddar, S. & Cate, J. H. D. Crispr–cas9 genome engineering in Saccharomyces cerevisiae cells. *Cold Spring Harb. Protoc.* **2016,** 525–533 (2016).

2. Montague, T. G., Cruz, J. M., Gagnon, J. A., Church, G. M. & Valen, E. CHOPCHOP: A CRISPR/Cas9 and TALEN web tool for genome editing. *Nucleic Acids Res.* **42,** 401–407 (2014).
